# Real-time decision-making during emergency disease outbreaks

**DOI:** 10.1371/journal.pcbi.1006202

**Published:** 2018-07-24

**Authors:** William J. M. Probert, Chris P. Jewell, Marleen Werkman, Christopher J. Fonnesbeck, Yoshitaka Goto, Michael C. Runge, Satoshi Sekiguchi, Katriona Shea, Matt J. Keeling, Matthew J. Ferrari, Michael J. Tildesley

**Affiliations:** 1 Department of Life Sciences, University of Warwick, Coventry, United Kingdom; 2 Mathematics Institute, Zeeman Building, University of Warwick, Coventry, United Kingdom; 3 CHICAS, Lancaster University, Bailrigg, Lancaster, United Kingdom; 4 Department of Infectious Disease Epidemiology, School of Public Health, Faculty of Medicine, St Mary’s Campus, Imperial College London, London, United Kingdom; 5 Department of Biostatistics, Vanderbilt University, Nashville, Tennessee, United States of America; 6 Center for Animal Disease Control, University of Miyazaki, Miyazaki, Japan; 7 Department of Veterinary Sciences, Faculty of Agriculture, University of Miyazaki, Miyazaki, Japan; 8 US Geological Survey, Patuxent Wildlife Research Center, Laurel, Maryland, United States of America; 9 Center for Infectious Disease Dynamics, Department of Biology, Eberly College of Science, The Pennsylvania State University, Pennsylvania, United States of America; 10 Department of Biology and Intercollege Graduate Degree Program in Ecology, 208 Mueller Laboratory, The Pennsylvania State University, Pennsylvania, United States of America; Emory University, UNITED STATES

## Abstract

In the event of a new infectious disease outbreak, mathematical and simulation models are commonly used to inform policy by evaluating which control strategies will minimize the impact of the epidemic. In the early stages of such outbreaks, substantial parameter uncertainty may limit the ability of models to provide accurate predictions, and policymakers do not have the luxury of waiting for data to alleviate this state of uncertainty. For policymakers, however, it is the selection of the optimal control intervention in the face of uncertainty, rather than accuracy of model predictions, that is the measure of success that counts. We simulate the process of real-time decision-making by fitting an epidemic model to observed, spatially-explicit, infection data at weekly intervals throughout two historical outbreaks of foot-and-mouth disease, UK in 2001 and Miyazaki, Japan in 2010, and compare forward simulations of the impact of switching to an alternative control intervention at the time point in question. These are compared to policy recommendations generated in hindsight using data from the entire outbreak, thereby comparing the best we could have done at the time with the best we could have done in retrospect.

Our results show that the control policy that would have been chosen using all the data is also identified from an early stage in an outbreak using only the available data, despite high variability in projections of epidemic size. Critically, we find that it is an improved understanding of the locations of infected farms, rather than improved estimates of transmission parameters, that drives improved prediction of the relative performance of control interventions. However, the ability to estimate undetected infectious premises is a function of uncertainty in the transmission parameters. Here, we demonstrate the need for both real-time model fitting and generating projections to evaluate alternative control interventions throughout an outbreak. Our results highlight the use of using models at outbreak onset to inform policy and the importance of state-dependent interventions that adapt in response to additional information throughout an outbreak.

## Author summary

Mathematical and simulation models may be used to inform policy in the early stages of an infectious disease outbreak by evaluating which control strategies will minimize the impact of the epidemic. In these early stages, significant uncertainty can limit the ability of models to provide accurate predictions, and policymakers do not have the luxury of waiting for data to alleviate this state of uncertainty. For policymakers, however, what is most important is the selection of the optimal control intervention, rather than accuracy of model predictions. We fit an epidemic model to observed, spatially-explicit, infection data at weekly intervals throughout two historical outbreaks of foot-and-mouth disease, UK in 2001 and Miyazaki, Japan in 2010, and compare forward simulations of the impact of alternative control interventions. These are compared to policy recommendations generated in hindsight using data from the entire outbreak. Our results show that the optimal control policy is identified accurately from an early stage in an outbreak, despite high levels of uncertainty in projections of epidemic size, and that the relative performance of control strategies is strongly mediated by our understanding of the locations of infected farms, rather than improved estimates of transmission parameters.

## Introduction

The responsibilities of policymakers during infectious disease outbreaks include the difficult task of choosing between multiple control interventions based on an uncertain future. Mathematical and simulation models have proved a useful tool to aid decision-making during disease outbreaks by both generating forecasts of outbreak severity and comparing different control strategies [1–6]. Using mathematical and simulation models, however, requires estimation of model parameters, and in the early stages of an outbreak, when decisions are most critical and data are most scarce, significant parametric uncertainty may limit the ability of models to provide informed advice.

Recent research has outlined frameworks that combine Bayesian parameter estimation and a mathematical model for generating real-time forecasts of outbreak severity throughout the course of an epidemic, aiming to assimilate available surveillance data into model estimates as rapidly as possible [7–10]. The efficacy of these frameworks has typically been evaluated using the accuracy of forecasts. For decision-makers, however, the accuracy of model forecasts per se, are not the best measure of success, it is the selection of the optimal control interventions in the face of uncertainty that counts, and ultimately what they are judged on. However, projections of the impact of alternative control interventions are not always performed alongside projections of the burden of infection. For several infectious diseases it is not possible, or relevant, to include projections of control interventions, for instance when interventions are directly related to estimates of the burden of disease or when control interventions are related to the timing of the peak of an epidemic (such as in influenza). Including projections of interventions does allow disease control problems to be phrased as optimization problems and therefore allows the determination of whether optimal control choice is dependent upon the underlying state of the outbreak. In contrast to real-time forecasting approaches, we demonstrate real-time decision-making, which requires integration of all information until now plus the potential future impact of candidate control interventions.

In this work, we simulated the process of real-time decision-making by fitting a dynamic epidemic model to the observed (confirmed), herd-level, infection data at weekly intervals throughout two historical outbreaks of foot-and-mouth disease (FMD), a viral disease of economically important livestock, and compared forward simulations of the impact of alternative culling and vaccination interventions under a management objective of minimizing total culls so as to gain disease-freedom. We repeated these forward simulations at each time point using parameter estimates from a model fitted to the complete outbreaks, thereby comparing the best we could have done at the time with the best we could have done in retrospect. Forward simulations predicted the final total culls (number of animals culled) from having taken a single control intervention from a particular date into the future. Control interventions included culling of infected premises only (IP), culling of infected premises and dangerous contacts (IPDC), culling of infected premises, dangerous contacts and contiguous premises (IPDCCP), ring culling in areas surrounding infected premises at 3 and 10 km radii (RC3 and RC10 respectively), and vaccination in areas surrounding infected premises at 3 and 10km radii (V3 and V10 respectively) (see Materials and Methods for further details of the control interventions). This set of intervention strategies includes those that governments have implemented, or considered, in the past and that are consistent with other studies on foot-and-mouth disease.

In contrast to previous work in real-time forecasting [7–10], we used an individual-based model and included uncertainty regarding the location of infected and undetected farms [11,12]. As surveillance data become available, we improve our understanding, in a cumulative manner, of both how the outbreak is unfolding, via estimates of transmission parameters, and of where likely new infections may be located, via estimates of the spatial distribution of infected but undetected farms. Forward simulations are therefore conditional upon both the actual state of the outbreak (i.e. the pattern of confirmed infected cases and the pattern of inferred, undetected infections) and having enacted the actual outbreak controls until the time point in question.

The outbreaks of FMD in the UK in 2001 and Miyazaki, Japan in 2010 contrast in a number of ways and thus provide a valuable comparison for investigating how policy recommendations are affected by additional information throughout an outbreak. Firstly, the UK outbreak had over 2000 infected premises over an area that included England, Wales, and Scotland (roughly 230000km^2^) [13] whereas the outbreak in Miyazaki affected only 290 premises contained within the Miyazaki prefecture (less than 8000km^2^) [14,15]. Secondly, at the time of confirmation of FMD, there were multiple foci of infection dispersed throughout the UK but only one focus of infection in the Miyazaki outbreak (although additional foci occurred later). Finally, control interventions deployed during each outbreak were different; UK control interventions only included culling strategies whereas control of the outbreak in Miyazaki began with culling and shifted to vaccination after 5 weeks, necessitated by constraints on the disposal of accumulating carcasses [16].

## Results

**Fig 1:**
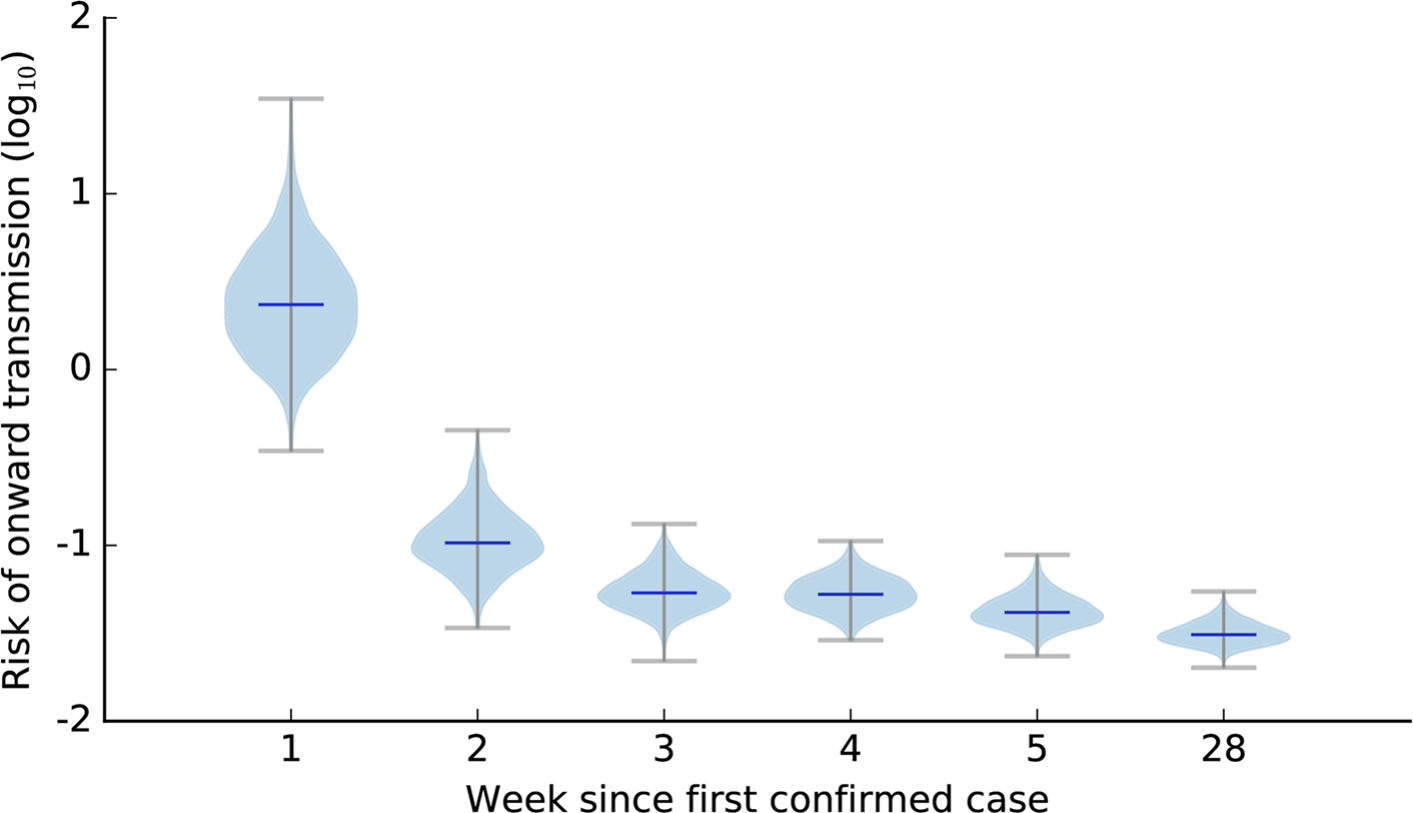
Instantaneous risk of onward transmission of foot-and-mouth disease in UK 2001 in first 5 weeks and the final week. Calculated as the infectious pressure from an average-sized infectious farm to an average-sized susceptible farm integrated across both the joint parameter distribution at the time point in question, and from 0 to 20km. Note that the instantaneous risk of transmission indicates the overall relative risk of transmission, which does not have a direct epidemiological interpretation but provides a direct comparison across weeks.

In the early stages of both the UK and Miyazaki outbreaks, estimates of the instantaneous risk of onward transmission are highly uncertain (Figs 1, S3 and S4). Our analysis highlights that uncertainty reduces through time in several regards. Firstly, estimates of transmission parameters change through time (Figs 1, S3 and S4). Secondly, our understanding of how transmission parameters relate to one another change through time (S5 and S6 Figs). Finally, our confidence in the locations of premises we believe to be infected also changes through time (Figs 1, S7 and S8). Given that the relationships between parameters change through time, marginal distributions of parameters (S3 Fig) do not tell the whole story, and we therefore summarize how the shape of the multidimensional posterior distribution evolves through time via a measure of instantaneous risk of onwards transmission. Note that the instantaneous risk of transmission indicates the overall relative risk of transmission, which does not have a direct epidemiological interpretation but provides a direct comparison across weeks.

**Fig 2:**
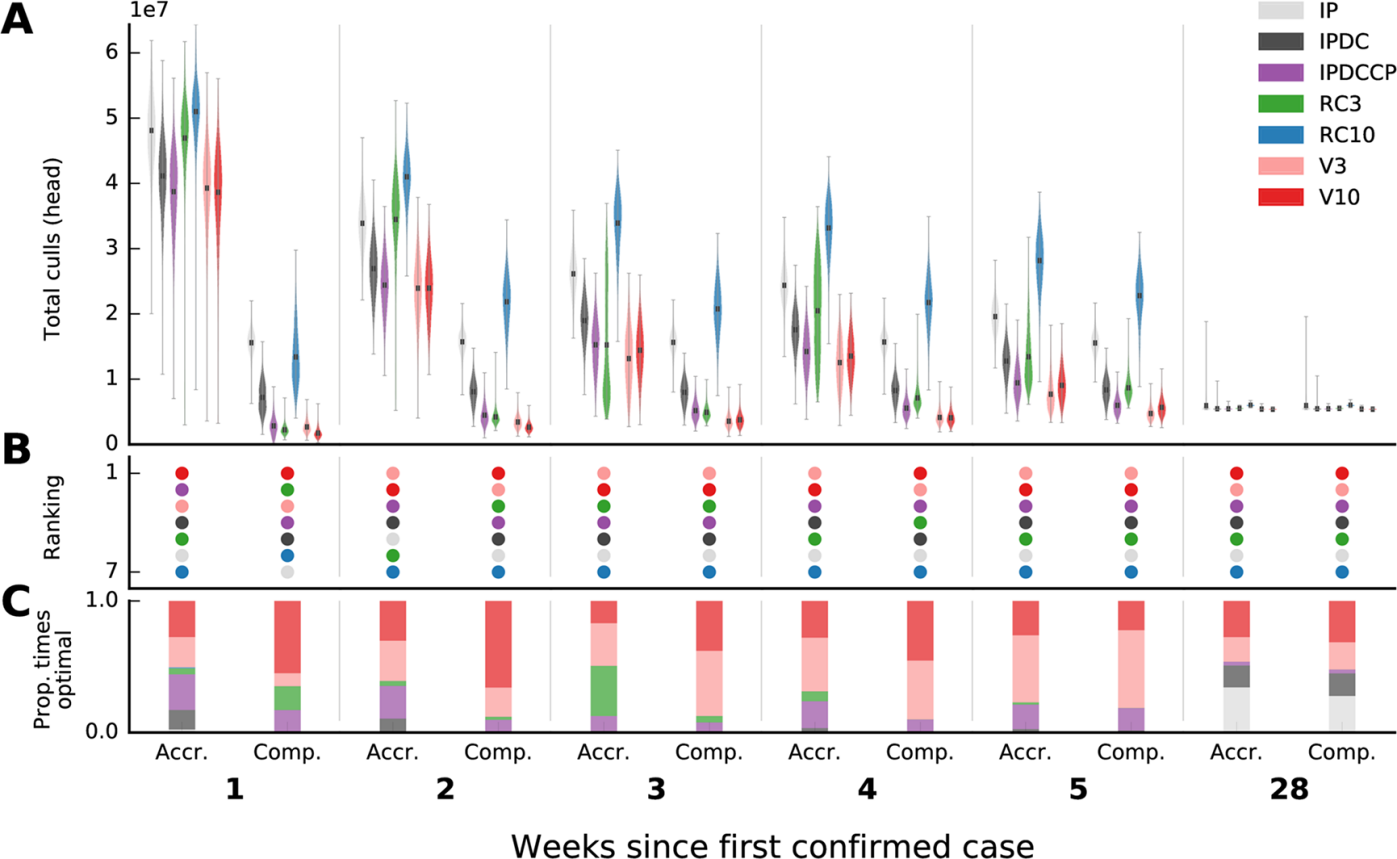
Projections and relative rankings of various control strategies of total animals culled, and estimates of infected but undetected farms, for the first five weeks and the final week of the 2001 foot-and-mouth disease outbreak in UK. A) Distribution of total animal culls from forward simulations, here shown as kernel density estimates (violin plots), are seeded either using parameter estimates from the end of the outbreak (Comp.; ‘complete’), or at the specific time point (Accr.; ‘accrued’). B) Rankings of control interventions are according to mean projections. Proportion (C) of times each control was optimal when bootstrap samples are made from distributions in (A). For all time points see S9 and S10 Figs.

**Fig 3:**
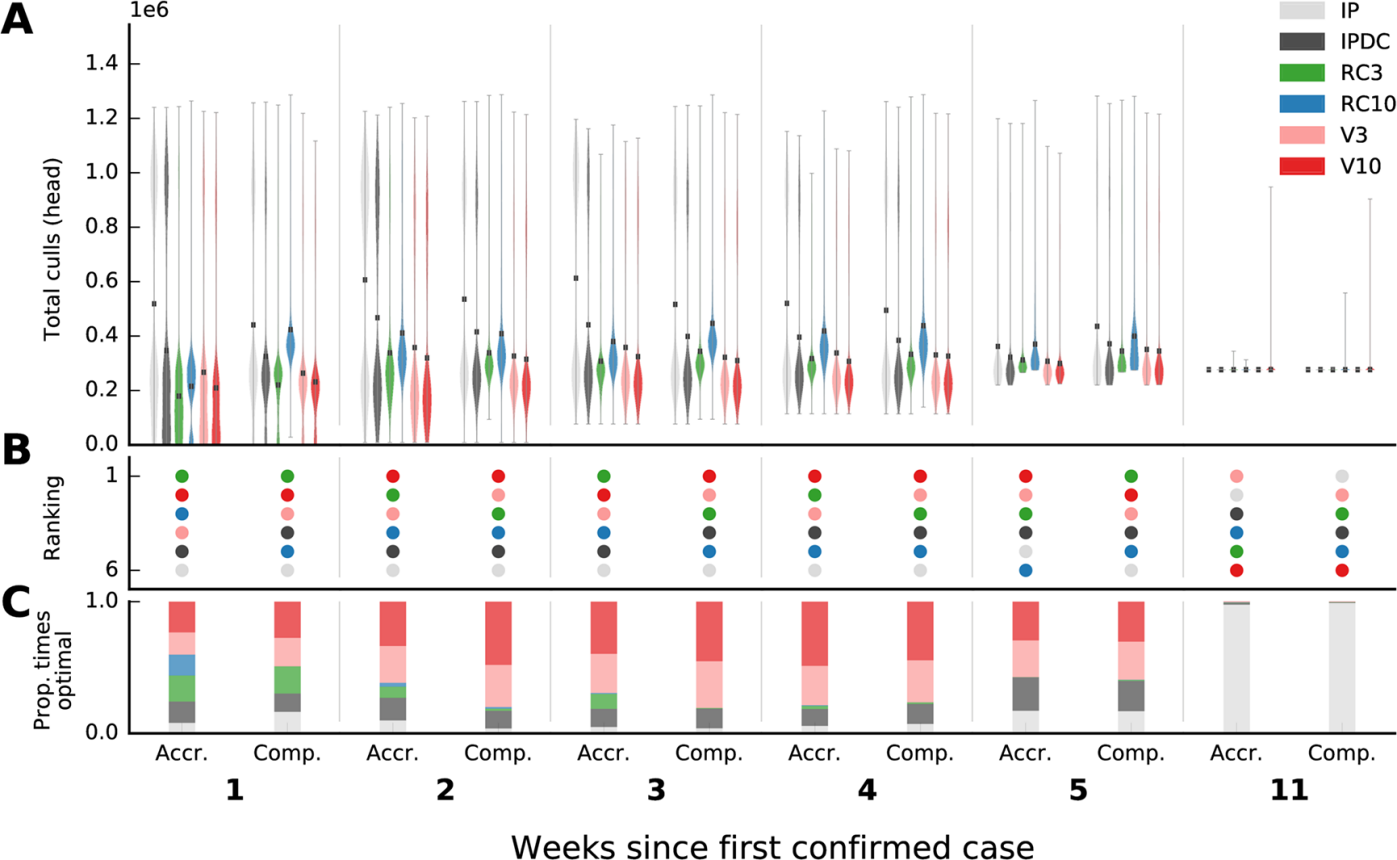
Projections and relative rankings of various control strategies of total animals culled, and estimates of infected but undetected farms, for the first five weeks and final week of the 2010 foot-and-mouth disease outbreak in Miyazaki, Japan. A) Distribution of total animal culls from forward simulations, here shown as kernel density estimates (violin plots), are seeded either using parameter estimates from the end of the outbreak (Comp.; ‘complete’), or at the specific time point (Accr.; ‘accrued’). B) Rankings of control interventions are according to mean projections. Proportion (C) of times each control was optimal when bootstrap samples are made from distributions in (A). For all time points see S11 Fig.

Projections of the final total number of animals culled that were made in the first three weeks of each outbreak were highly uncertain, grossly overestimating the size of the outbreak in the UK and, on average, tending to underestimate the size of the outbreak in Miyazaki (Figs 2 and 3). In the UK, the mean outbreak size for vaccination strategies was estimated to be ten times larger when using one week’s worth of data compared to when using all of the data (Fig 2; accrued vs complete, week 1). In Miyazaki, bimodal distributions of outbreak size were generated by forward simulations started in the initial weeks as the spatial extent of the outbreak was highly uncertain (Fig 3). This was particularly marked under strategies of non-ring control culling (IP and IPDC). However, in both outbreaks, despite projections being highly variable, the relative performance of control interventions was resolved early on (Figs 2B and 3B). The projected rankings of control interventions between those based on available data and those based on all outbreak data were identical at five weeks into the UK epidemic (S9 and S10 Figs), and differences in the rankings of control interventions after week 5 were minor. Using only available data, the top three ranked control strategies (vaccination at 3km, 10km, and IPDCCP culling, respectively) do not change from weeks 5–8, at which point vaccinating at 10km becomes optimal over vaccinating at 3km in week 9. Vaccination interventions (either at 3km or 10km) are always ranked as optimal throughout the whole UK outbreak regardless of the week at which projections were made and regardless of the amount of data available. If vaccination was deemed politically unpalatable then culling of infected premises, dangerous contacts, and contiguous premises (IPDCCP) was consistently ranked as the next best intervention to minimize total culls from weeks 4–10 of the outbreak in the UK (regardless of whether available or all data were used to estimate transmission parameters). Although the rankings of control interventions did change in the Miyazaki outbreak, the relative distribution of expected outbreak sizes across different control interventions remained consistent, with 3km and 10km vaccination strategies consistently ranked as optimal (Figs 3 and S11) and consistently performing better than the other strategies when compared in repeated bootstrap simulations (Figs 3C and S11C). Ring culling at 3km was ranked as optimal for weeks 1–5 if vaccination interventions were not considered, regardless of the amount of data used to estimate transmission parameters. IP culling was optimal in the final stages of both outbreaks when there were few or no more infected premises (S1, S2 and S10 Figs). The consistency in the relative rankings of control interventions lends support for trusting model comparisons of interventions at outbreak onset.

For the UK outbreak on week 2 (5 March 2001), when only using available data, projections of the magnitude of the four best performing control interventions occupied comparable overlapping ranges: V3, V10, IPDC and IPDCCP (accrued information; Fig 2). Such a set of projections may provide a strong case in support of only enacting IPDC, the minimum under EU and Japanese law, as this avoids the latent underlying costs and consequences of culling contiguous premises or emergency vaccination, for only a small increase in the expected number of total animals culled. However, had all the data for the outbreak been available at this date (complete information; Fig 2), then the expected performance of IPDC was revealed to be very poor compared to these other three control interventions, with the results supporting either vaccination (V3 or V10) or culling in some proximity around infected farms (RC3 or IPDCCP) to quickly halt the outbreak. Here, despite variability in the absolute value of projections, our initial rankings and estimates of the relative performance of controls were again found to be robust.

In the outbreak in Miyazaki the three best control interventions were correctly identified by week 2 using only the available data despite highly uncertain projections of total animals culled (Figs 3 and S11). Relative performance of control interventions was largely unchanged after this point, with the exception of the emergence of IP culling as optimal in the final weeks due to there only having been 1 infected case on 4th July, before which there had not been a case since 18 June 2010 [14]. Consistency in the performance of vaccination strategies is highlighted by noting that greater than 50% of the time vaccination actions are optimal when bootstrap samples are compared across the distributions of simulated total culls for weeks 2–5 (Figs 3C and S11C). Bimodal distributions in outbreak size did reappear later in the outbreak between weeks 6–7 (1–8 June) coincident with the occurrence of additional foci of infection (S11 Fig), although these additional outbreaks were effectively controlled and policy recommendations were unchanged. This is in contrast to the UK 2001 outbreak where, at the time of the first case being reported, there were already infected farms in multiple foci spread across the country with no significant new emerging foci as the epidemic progressed that would give rise to the bimodal predictions that we observe for Miyazaki. Overall, outbreak size and optimal control choice for the Miyazaki outbreak were generally more straightforward to predict than for the UK outbreak, and policy recommendations for minimizing outbreak size are in line with what was actually implemented in 2010 (vaccination at a 10km radius, V10).

Changes in policy recommendations and large uncertainty in model projections may be caused by uncertainty associated with the parameters governing the dynamics of the outbreak and/or uncertainty associated with the locations of infected farms. By looking at projections made using parameters generated with complete outbreak data, isolating the effect of changing the arrangement of infected farms (since transmission parameters are fixed in these simulations), we see that policy recommendations are still changing. Between 5 and 12 March 2001 in the UK outbreak (weeks 2–3), there was a marked resolution in the performance of control interventions when parameters were estimated using only available data (accrued information). The relative performance of control interventions at this time point comes to resemble analogous projections made using all outbreak data (Figs 2, S9 and S10) despite the absolute value of such projections still being highly uncertain. Here, this switch may have been caused by a constrained estimate of where the outbreak is–an improved estimate of the location of undetected infected premises (S7 Fig). Our best estimate of the location of undetected infections on 5 March 2001 (week 2) includes undetected infections in the north of Scotland. Additionally, the number of counties with a non-zero expected number of undetected infections is overestimated compared to analogous estimates if all the data were available (S7 Fig). Our best estimate of undetected infections on 12 March 2001 (week 3) had foci of infection in Cumbria, Midlands, Devon, North Wales, and Essex; these foci were in the same areas as estimates using all outbreak data. Furthermore, those counties that have a non-zero expected number of undetected infections are contiguous to counties that are estimated as a foci of infection (S7 Fig). At this point, by excluding an allowance for additional culling in Scotland, ring culling at 3km became second only to vaccination actions. Ultimately, the change in our understanding of the locations of infected farms (and having the resource capability to respond), drove this improved prediction of the relative performance of control interventions.

Such a dramatic change in the relative performance of control interventions was not seen in the Miyazaki outbreak, despite starting with high variability in projections of the efficacy of control interventions. The Miyazaki outbreak started and largely stayed in one location, the Tsuno township. Though bimodal distributions in outbreak size did arise in simulations (S11 Fig) as additional foci of infection occurred later at the Ebino township, Miyakonojo, and Miyazaki city (all over 40km away from the original point of infection [17]), the projections were largely seeded by an outbreak with a state characterized as a single point of infection, with compact and radial spread of infection (Figs 3, S8 and S11). Accounting for the possibility of a larger outbreak size in Miyazaki did not stretch the simulated culling or vaccination resources, leaving policy recommendations for how we should respond unchanged. As with results from the UK outbreak, uncertainty associated with the locations of undetected infected premises, in the form of additional foci of infection, seemed to have a large effect on forward projections in the Miyazaki outbreak.

## Discussion

Our results have shown that policy recommendations using real-time parameter estimates and forward simulations can be correct from an early stage in an outbreak despite highly uncertain projections of epidemic severity, in line with previous research on one-time decisions for the control of Ebola [18]. Projections from mathematical and simulation models are useful for informing policy-making insofar as they help to identify what is the best course of action and we have included two summaries of how simulation results may be communicated, dependent upon whether policymakers have an objective of 1) optimizing an expectation in outbreak severity (Figs 2B and 3B) or 2) optimizing the number of times that an intervention was predicted to be optimal (Figs 2C and 3C). In many situations, estimating the burden of infection and selection of the optimal control intervention are directly related. However, our results are a reminder that this may not always be the case; in many cases it is how projections influence change in the recommended control intervention, rather than the value of projections themselves, that matter and so, where appropriate, it is important to include projections of intervention efficacy when making real-time projections of disease burden.

Our results showed that changes in the estimated relative performance of control interventions was strongly influenced by the spatial distribution of both observed and undetected infectious premises and culled premises (the state of the outbreak). Although our analysis only looked at one change in control intervention (from what was being applied historically) this dependence highlights that, should one allow multiple changes of control through time, then the optimal policy is likely to be dynamic. In our results, even if accurate parameter estimates had been available from the first confirmed case (such as those derived from the whole outbreak), it would still have been necessary to perform state-dependent control to find the best choice of control intervention as the outbreaks evolve. In practice, estimating the state of the outbreak (i.e. prediction of locations of undetected infections) depends on estimates of transmission parameters and, therefore, continual re-evaluation of control interventions alone makes little sense without also re-evaluating, and re-fitting, model parameters. Thus, control recommendations should adapt to the changing state of an outbreak.

Our results included a marked stabilization in recommended control interventions in simulations for the outbreak in the UK. It remains an avenue for future research to investigate what are the drivers of such stabilization in recommendations in control intervention, and whether it is logistically feasible to measure (and therefore act upon) such drivers, beyond an in silico experiment. Such analyses would require a more general approach to seeding outbreaks, and generating landscapes, to more thoroughly explore the state-space of potential future outbreaks. Here, we only investigated two outbreaks, simulations for each of which were seeded from historical starting points.

Often, the need for rapid response during an outbreak is discussed in terms of the initial response [13], but not as often in terms of adapting as circumstances change throughout an outbreak. In both outbreaks we investigated, there was significant variability in epidemiological predictions during the early stages of disease outbreaks highlighting that real-time updating of parameters is vital in order to obtain accurate predictions of epidemic size and extent. However, additional foci of infection may be seen as independent outbreaks and, therefore, the rapid response that is called for at the start of an outbreak needs to be reapplied when such foci are discovered. For the outbreaks that we investigated, identifying the locations of undetected infected premises was of greater importance to determining the best course of action than identifying the underlying disease dynamics; this calls for increased vigilance in surveillance during an outbreak and highlights the importance of methodology for predicting undetected infections (e.g. [11,12]), assimilating surveillance data into parameter estimates for individual-based models, and of confirming negative cases, especially when such premises are in locations that would be considered as additional foci of infection. Simply because an outbreak has progressed beyond its initial stages does not mean that the need to act swiftly according to changes in the outbreak are in any way diminished.

At a coarse level, for one-time decisions, the idea of state-dependent control has already been adopted or discussed by several agencies, such as the Department for Environment, Food, and Rural Affairs, UK (DEFRA) and the United States Department of Agriculture (USDA) in flowcharts for determining when to perform emergency vaccination [19,20], the dependence of different phases in smallpox eradication upon smallpox prevalence [21], management of wildlife diseases [22], and the use of adaptive surveillance of herds in the eradication of rinderpest [23]. Decision-making frameworks, such as adaptive management, have also highlighted the utility of modeling and optimization as tools for generating state-dependent policies [24]. We note that optimization methods such as dynamic programming or reinforcement learning would be required to generate state-dependent policies and that such methodologies, although a more complex optimization than what we have presented, would allow the possibility of changing control intervention repeatedly through time, or generating optimal interventions that are a combination of those defined here, such as county-specific interventions (e.g. [25]). Our analysis shows there is potential to make large gains in the effectiveness of the response to an outbreak by adopting state-dependent control that is on a much more nuanced scale than one-time binary decisions, and that mathematical and simulation models can play a significant role in policy preparedness by investigating such strategies before an outbreak occurs.

## Materials and methods

We used data augmentation Markov chain Monte Carlo (MCMC) to fit a 3-species (cattle, sheep, pigs) SEINR (Susceptible-Exposed-Infected-Notified-Removed) model to the observed infection and intervention data at the farm-level at weekly time points throughout the epidemics [11,12]. The analysis started one week after the first confirmed case (one week after 19 February 2001 and 20 April 2010 for the outbreaks in UK and Miyazaki respectively). Data for the UK included location and farm demography for approximately 188000 premises. Data for Miyazaki included location and farm demography for approximately 9000 premises. In the case of the UK outbreak, we changed the timescale to 28-day time points after 11 June 2001 until 3 September 2001. A final model fit represents all information that could have been garnered from the outbreak (on 24 December 2001 for the UK outbreak and 6 July 2010 for the outbreak in Miyazaki). Each model fit included an estimate of a joint posterior distribution of 16 parameters (only 12 parameters had changing estimates in Miyazaki since no sheep were affected in this outbreak) and an estimate of the infection time of infected, but as yet undetected, premises (so-called ‘occult’ infections). To reproduce a comparable level of initial uncertainty, the same uninformative prior distributions were used for each parameter at the start of the two outbreaks, while a uniform prior was used for the initial infection times of the undetected but infected farms.

At each time point, parameter estimates were used within a previously-validated farm-level simulation model [1,26], to make forward projections under various control interventions. Each set of parameter values in the posterior distribution also had an associated set of infection times of undetected infections. These were combined with the confirmed infections from the real outbreak data and used to seed the infectious status of each farm in simulations. Predictions were therefore conditional on the actual state of the outbreak at that point in time as well as our estimate of parameters and which farms were infected. Forward projections were made twice for each time point: once with parameter estimates (and the associated distribution of infection times of undetected infections) based only upon the data available at that time point, denoted ‘accrued information’; and once with analogous estimates made using all the data from the entire outbreak, denoted ‘complete information’.

Six (Miyazaki) or seven (UK) control interventions were compared in the forward simulations (Table 1). The performance of culling infected premises only (IP) was included as the least severe strategy possible, and the culling of infected premises and dangerous contacts only (IPDC) was included as the minimum required under European Union [27] and Japanese [28] law. In the UK simulations, the control of culling contiguous premises of infected farms (IPDCCP) was also included beyond controls used in the simulations for Miyazaki; this is most similar to that which was implemented during the 2001 outbreak [1]. Simulations for both outbreaks included ring culling around infected premises at 3km (RC3) and 10km (RC10) radii. Two emergency vaccination strategies, only targeting cattle, were also included at 3km and 10km around infected premises (V3 and V10 respectively), and were assumed to be vaccinate-to-live policies; where vaccinated cattle enter the food chain and are not included in counts of total animals culled. Note that a vaccinate-to-live policy was not applied in the outbreak in Miyazaki but it may be a future possibility in the UK [19]. These vaccination radii were used because 1) they aided in comparison with ring culling interventions; 2) they spanned a range that includes the optimal ring radius for reactive vaccination in the UK [4], and; 3) they included the radius (10km) at which vaccination was actually implemented in Miyazaki [14]. All parameter estimate data and simulation output are available via the Dryad Digital Repository: https://doi.org/10.5061/dryad.gr656gk [29].

**Table 1:** Summary of control interventions used in the simulations.

Abbreviation	Description	Constraints	Case study
IP	Culling of infected premises only	No constraints	UK and Miyazaki
IPDC	Culling of infected premises and dangerous contacts	100 farms per day	UK and Miyazaki
IPDCCP	Culling of infected premises, dangerous contacts, and premises contiguous to infected premises	100 farms per day	UK only
R3	Ring culling at 3km radius around infected premises (IPDC included)	300 farms per day	UK and Miyazaki
R10	Ring culling at 10km radius around infected premises (IPDC included)	300 farms per day	UK and Miyazaki
V3	Vaccination at 3km radius around infected premises (IPDC included)	300 farms per day or 30,000 animals (whichever is first)	UK and Miyazaki
V10	Vaccination at 10km radius around infected premises (IPDC included)	300 farms per day or 30,000 animals (whichever is first)	UK and Miyazaki

### Model

Both outbreaks used a similar model structure. The infectious pressure, *λ*_*j*_*(t)*, on a susceptible farm *j* at time *t* is

$$\lambda_{j}(t)=\epsilon (t)+\sum_{i\in I(t)}\beta_{ij}h(I_{j}-I_{i})+\sum_{i\in N(t)}\beta_{ij}^{*}h(I_{j}-I_{i}),$$

where I(t) and N(t) are the sets of infected and notified farms at time *t* respectively (*t = 0*, *1*, *2*, *… T*). Models of similar structure have been previously used for modeling FMD (e.g. [1,4,11]). A summary of notation used to describe the model is given in Table 2. Notification time is assumed to mean the time of laboratory confirmation of FMD, and removal time is assumed to mean the date when the farm is both culled and disposed of. We count *t* in days; day 0 is the first confirmed infected case in the data and *T* is the day of the final parameter estimate (24 December 2001 for the UK outbreak and 6 July 2010 for the outbreak in Miyazaki). By including both S(t) and R(t), as the sets of susceptible and removed farms at time *t* respectively, we then have four sets essentially giving the state of the epidemic on the population at a given point in time, $S_{t}=\langle S(t),I(t),N(t),R(t)\rangle$. The infection time of the *k*th farm is denoted *I*_*k*_, while *N*_*k*_ and *R*_*k*_ denote notification and removal times of farm *k* respectively. Note that in the next section we assume that for *I*_*t*_, *N*_*t*_, and *R*_*t*_, the *t* subscript denotes the set of infection, notification, or removal times of all premises respectively; here the time subscript is dropped for succinctness. All times are listed in days unless otherwise specified. Infectious pressure can be decomposed into contributions from infectious (but not yet notified) farms,

$$\beta_{ij}=\gamma_{1}q(i;\xi )w(j;\zeta )\frac{\delta }{{\left(\delta^{2}+\rho_{ij}^{2}\right)}^{\omega }},\;i\in I(t),j\in S(t),$$ (1)

and contributions from notified farms $\beta_{ij}^{*}=\gamma_{2}\beta_{ij}\;(i\in N(t),j\in S(t))$. We define

$$q(i;\xi )=\left[{\left(\frac{c_{i}}{\bar{c}}\right)}^{\psi_{1}}+{\xi_{2}(\frac{p_{i}}{\bar{p}})}^{\psi_{2}}+\xi_{3}{\left(\frac{s_{i}}{\bar{s}}\right)}^{\psi_{3}}\right]$$

and

$$w(j;\zeta )=\left[{\left(\frac{c_{j}}{\bar{c}}\right)}^{\phi_{1}}+{\zeta_{2}(\frac{p_{j}}{\bar{p}})}^{\phi_{2}}+\zeta_{3}{\left(\frac{s_{j}}{\bar{s}}\right)}^{\phi_{3}}\right]$$

to be the ‘infectivity’ of farm *i* and ‘susceptibility’ of farm *j* respectively, where *c*_*k*_, *p*_*k*_, and *s*_*k*_ are the numbers of cattle, pigs, and sheep on farm *k*, with mean numbers of cattle, pigs, and sheep, denoted by $\bar{c},\;\bar{p},\;\bar{s}$. The effect of distance is captured using a Cauchy-type kernel where *ρ*_*ij*_ is the Euclidean distance between farms *i* and *j*, and *δ* the decay of transmission rate with distance. Baseline infectious pressure distinguishes between periods before and after the movement ban was implemented,

$$\epsilon (t)=\left\{ \begin{array}{l} \epsilon_{1}\;t<movement\;ban\\ \epsilon_{1}\epsilon_{2}\;otherwise \end{array}\right.,$$

and the latency of the disease was modeled using the function

$$h(t)=\left\{ \begin{array}{l} 1\;t<4\\ 0\;otherwise. \end{array}\right.$$

An estimate at time *t* of the complete parameter set, of 16 parameters (12 parameters in Miyazaki), is denoted ***θ***_***t***_. Parameter *ω* was assumed fixed at 1.3 in both outbreaks, as were the initial priors. The priors for each parameter were elicited via expert opinion and assigned the following distributions: *π*_0_(*δ*)∼*Gamma*(4,8),*π*_0_(*ϵ*_1_),*π*_0_(*ϵ*_2_)∼*Gamma*(1*E*−7,1), *π*_0_(*ψ*_1_),*π*_0_(*ψ*_2_),*π*_0_(*ψ*_3_),*π*_0_(*ϕ*_1_),*π*_0_(*ϕ*_2_),*π*_0_(*ϕ*_3_),*π*_0_(*ξ*_2_),*π*_0_(*ξ*_3_),*π*_0_(*ζ*_2_),*π*_0_(*ζ*_3_) ∼*Gamma*(0.5,1),*π*_0_(*γ*_1_),*π*_0_(*γ*_2_)∼*Gamma*(1*E*−4,1). *Gamma*(*a*, *b*) is the gamma distribution with shape parameter *a* and rate parameter *b*.

**Table 2:** Summary table of mathematical symbols used.

Symbol	Description	Notes
*λ*_*j*_(*t*)	Infectious pressure on susceptible farm *j* at time *t*	
*β*_*ij*_	Contribution to total infectious pressure from infectious farms.	
$\beta_{ij}^{*}$	Contribution to total infectious pressure from notified farms.	$\beta_{ij}^{*}=\gamma_{2}\beta_{ij}$
S(t),I(t), N(t), R(t)	Set of susceptible, infected, notified, and removed farms (respectively) at time *t*.	
$S_{t}$	Complete state of the outbreak at time *t*.	$S_{t}=\langle S(t),\;I(t),\;N(t),R(t)\rangle$
*h*(*t*)	Function for modeling disease latency.	$h(t)=\left\{ \begin{array}{l} 1\;t<4\\ 0\;otherwise. \end{array}\right.$
*I*_*k*,_ *N*_*k*,_ *R*_*k*_	Infection, notification and removal times of farm *k* (respectively).	Notification time is time of laboratory confirmation of FMD; removal time is mean date when the farm is both culled and disposed of.
*ϵ*(*t*)	Baseline infectious pressure at time *t*.	$\epsilon (t)=\left\{ \begin{array}{l} \epsilon_{1}\;t<movement\;ban\;\\ \epsilon_{1}\epsilon_{2}\;otherwise \end{array}\right.$
*q*(*i*;***ξ***)	Contribution to infectious pressure from characteristics of the infectious farm.	***ξ*** = {*ξ*_2_,*ξ*_3_}
*w*(*j*;***ζ***)	Contribution to infectious pressure from characteristics of the susceptible farm.	***ζ*** = {*ζ*_2_,*ζ*_3_}
***ξ*** = {*ξ*_2_,*ξ*_3_}	Parameters for determining the transmissibility of pigs (2) and sheep (3) relative to cattle.	
***ζ*** = {*ζ*_2_,*ζ*_3_}	Parameters for determining the susceptibility of pigs (2) and sheep (3) relative to cattle.	
*ψ*_1_,*ψ*_2_,*ψ*_3_	Exponential terms for calculating infectious pressure from an infectious farm for cattle(1), pigs(2), and sheep(3) respectively.	
*ϕ*_1_,*ϕ*_2_,*ϕ*_3_	Exponential terms for calculating infectious pressure for a susceptible farm for cattle (1), pigs (2), and sheep (3) respectively.	
*γ*_1_,*γ*_2_	Multiplicative factor for contribution to infectious pressure from infected (1) and notified (2) farms respectively.	
*δ*	Decay of the transmission rate with distance between premises.	
*ρ*_*ij*_	Euclidean distance between premises *i* and *j*.	
*ω*	Exponential parameter associated with the distance kernel.	Fixed at 1.3 in all outbreaks.
***θ***_***t***_	Vector of all transmission parameters.	***θ*** = {*ϵ*_1_,*ϵ*_2_,*γ*_1_,*γ*_2_,***ξ***,***ζ***,***ψ***,***ϕ***,*δ*,*b*}
*c*_*k*_, *p*_*k*_, *s*_*k*_	Number of cattle, pigs and sheep (respectively) on premises *k*.	
$\bar{c},\;\bar{p},\;\bar{s}$	Mean numbers of cattle, pigs, and sheep (respectively) across all farms in the outbreak.	
*d*_*i*_	Infection to notification time.	*d*_*i*_ ~ Gamma (4, b)
*b*	Scale parameter of a gamma distribution governing infection to notification time.	Set at 0.5.
$X_{t^{-}}$	Demographic and event history data observed to time *t*.	
*Y*_*t*_	Demographic and event data of the ongoing epidemic from time *t* onwards.	
$\pi_{t}(\theta_{t},I_{t}|X_{t^{-}})$	Joint posterior distribution of model parameters and infection times at time *t*, conditional on the demographic and event history data.	
$I_{j}^{-}$	The time immediately before the infection time of the *j*th premises.	
***P***	Set of all individuals in the population.	
*κ*	The initial infective.	
*a*	Control intervention *a*.	
*U*(*Y*_*t*_|*a*)	Expected total number of animals culled (utility function) in the ongoing outbreak from time t onwards under intervention *a*.	
$a_{t}^{⋆}$	Optimal control strategy at time *t*.	

Infection to notification time was assumed to be distributed according to a Gamma distribution such that *N*_*i*_ → *I*_*i*_ = *d*_*i*_∼*Gamma*(4,*b*) with parameter *b = 0*.*5* governing the scale of the probability distribution. Notification to removal time is an observed quantity since both events are recorded.

### Parameter estimation

Let the data (demographics and event history) observed up to time *t* be $X_{t^{-}}$, with $\pi_{t}(\theta_{t},I_{t}|X_{t^{-}})$ denoting the joint posterior distribution of the model parameters and infection times (including infection times of undetected infections) immediately at time *t*.

We assume that infections occurred according to a continuous-time non-homogeneous Poisson process, where infection rate is assumed to be *λ*_*j*_*(t)* as above. Let ***I***, ***N***, and ***R*** be vectors of infection, notification, and removal times for individuals *1*, *…*, *n*_*I*_ who were infected by observation time *T*_*obs*_. Conditioning on this event time, the joint posterior distribution over parameters ***θ*** = {*ϵ*_1_,*ϵ*_2_,*γ*_1_,*γ*_2_,***ξ***,***ζ***,***ψ***,***ϕ***,*δ*,*b*} is

$$\pi (\theta |I,N,R)\propto \prod_{j=1}^{n_{I}}\left[\lambda_{j}(I_{j}^{-}\right)]exp\left[\int_{I_{\kappa }}^{T_{obs}}\sum_{j\in P}\lambda_{j}(t)dt\right]$$

$$\times \prod_{j=1}^{n_{I}}{\left(N_{j}-I_{j}\right)}^{a-1}e^{-b(N_{j}-I_{j})}$$

$$\times \prod_{p=1}^{\left|\theta \right|}f_{\theta_{p}}(\theta_{p}),$$

where the first line represents the infection process, the second line represents the detection (infected to notified) process, and the third line represents independent prior distributions for all components of ***θ***. $I_{j}^{-}$ represents the time immediately before the infection time of the *j*th premises.

Parameters in bold, represent the corresponding set of species-specific parameters, e.g. ***ζ*** = {*ζ*_2_,*ζ*_3_}. *κ* denotes the initial infective, and ***P*** denotes the set representing all individuals in the population. The joint posterior distributions used in the forward simulations and figures of the instantaneous risk of onward spread represent the MCMC output sub-sampled to 2000 parameter coordinates.

### MCMC algorithm

Multisite adaptive Metropolis-Hastings was used to draw from the conditional posterior distributions of {*ϵ*_1_,*ϵ*_2_,*γ*_1,_,*γ*_2_,*δ*}, ***ψ*** and ***ϕ***. Furthermore, since the infection times are unobserved, we updated them component-wise using Metropolis-Hastings, with a reversible-jump update to explore the posterior over the presence of undetected infections. See [11,12] for further details.

### Updating species effects ξ and ζ

The introduction of the 3-species model (in comparison to [11] and [1]) results in a non-linearly correlated posterior distribution, particularly between *γ*_1_ and both of ***ξ*** and ***ζ***. This presents particular difficulties for Metropolis-based MCMC algorithms, since the optimal proposal distribution scale changes with location in the posterior parameters space. To approximately orthogonalize the posterior distribution, and hence improve the convergence properties of our adaptive Metropolis algorithm, we employed the following non-centered update for ***ξ*** and ***ζ***.

To construct an efficient proposal distribution for ***ξ***, we seek to exploit the shape of the posterior distribution with respect to *γ*_1_. This can be thought of as a joint update of *γ*_1_ and ***ξ***, respecting the contour of the joint posterior. First, we write the equation for *q*(*i*;***ξ***) (see above) as

$$\gamma_{1}q(i;\xi )=\gamma_{1}C_{i}+\gamma_{1}\xi_{2}P{}_{i}+\gamma_{1}\xi_{3}S_{i}$$

$$=A_{\left\{i,1\right\}}+A_{\left\{i,2\right\}}+A_{\left\{i,3\right\}}$$

where $C_{i}={{(c}_{i}/\bar{c})}^{\psi_{1}}$ and similarly for *P*_*i*_ and *S*_*i*_. We then let

$$R=A_{1}+A_{2}+A_{3}=\sum_{i=1}^{n_{I}}{(A}_{\left\{i,1\right\}}+A_{\left\{i,2\right\}}+A_{\left\{i,3\right\}})$$

We then propose

$${\left(A_{2}^{*}+A_{3}^{*}\right)}^{T}∼MVN_{2}({\left(A_{2}+A_{3}\right)}^{T},{\frac{2.38}{2}}^{2}B)$$

and

$$A_{1}^{*}=R-A_{2}^{*}-A_{3}^{*}$$

where, with probability 0.05, *B = I*_*2*_ the 2x2 identity matrix, and with probability 0.95, B = *Σ*_*k*_ the empirical covariance matrix of the MCMC samples for (*A*_1_,*A*_2_)^*T*^ up to iteration *k* (see [30]). We then solve for *γ*_1_ and ***ξ***. Similarly, we update *γ*_1_ and ***ζ***.

### Instantaneous risk of onward spread

Uncertainty in the joint posterior distribution of transmission parameters was summarized using a measure of the instantaneous risk of onward spread, defined as the instantaneous force of infection from an average-sized infectious farm to an average-sized susceptible farm at time *t* (*g*_*t*_). This risk measure was calculated at each time point as the integral over the joint posterior distribution of parameters at that point in time and over a distance of 20km (from the infectious to the susceptible farm):

$$g_{t}=\int_{\theta }^{}\int_{0}^{20}\gamma_{1}[\xi_{2,t}+\xi_{3,t}][\zeta_{2,t}+\zeta_{3,t}]\frac{\delta_{t}}{{\left(\delta_{t}^{2}+\rho^{2}\right)}^{\omega }}d\rho d\theta .$$

The equation for the instantaneous risk of onward spread is taken from the equation for the infectious pressure (Eq 1) substituting the average number of cattle, pigs, and sheep for *c*_*i*_, *p*_*i*_, and *s*_*i*_ respectively.

### Forward simulations

Each control intervention, *a*, is evaluated according to the expected total number of animals culled, *U*(*Y*_*t*_|*a*), where the Bayesian predictive distribution of the ongoing epidemic, *Y*_*t*_, is given by the integral

$$f_{Y_{t}}(Y_{t}|X_{t^{-}},a)=\int_{\Theta }\int_{I}f_{Y_{t}}(Y_{t}|X_{t^{-}},\theta_{t},I_{t},a)\pi_{t}(\theta_{t},I_{t}|X_{t^{-}})dId\theta .$$

This was estimated by simulating forward from $f_{Y_{t}}(Y_{t}|X_{t^{-}},\theta_{t},I_{t},a)$ using draws from $\pi_{t}(\theta_{t},I_{t}|X_{t^{-}})$. The above formulation is using parameter estimates from the time point in question, so-called ‘accrued information’. Forward simulations were also generated with parameters estimated using all data, $f_{Y_{t}}(Y_{t}|X_{T^{-}},a)$, so-called ‘complete information’, which were generated in an analogous fashion instead using draws from $\pi_{T}(\theta_{t},I_{t}|X_{T^{-}})$ in the above integral.

The optimal control strategy $a_{t}^{⋆}$ is chosen as that which minimizes the mean expected total animals culled

$$a_{t}^{⋆}={argmin}_{a}mean[U(Y_{t}|a)]$$

Vaccine efficacy was assumed to be 90%, whereby animal numbers on a vaccinated farm were reduced by 90% after a delay until conferment of immunity of 4 days. Delay from infection to notification time was simulated as 9 days, with a 4-day latency period. Notification to removal time was simulated as 1 day for infected premises and 2 days for culling of dangerous contacts or contiguous premises.

### Software

The MCMC algorithm above was implemented in C++ (GCC version 4.8.3) embedded within an R package. General-purpose graphics processing unit (GPU) using NVIDIA CUDA 7.5 was used to implement the calculation of the likelihood and speed up inference. The software is available under the GPLv3 license at http://fhm-chicas-code.lancs.ac.uk/InFER/InFER/tags/InFERfmd-v1.0.

## Supporting information

S1 Fig Number of confirmed cases per week (ending Monday) during the 2001 outbreak in the UK. (TIF)

S2 Fig Number of confirmed cases per week (ending Tuesday) during the 2010 outbreak in Miyazaki. (TIF)

S3 Fig Marginal posterior predictive distribution of 16 parameters in the epidemic model for the first 11 weeks. Distributions in red are estimated for the outbreak in Miyazaki blue for parameters the UK. Parameters shown on the log scale are γ_1_, ε_1_, and ε_2_.
(TIF)

S4 Fig Instantaneous risk of onward transmission of foot-and-mouth disease in Miyazaki 2010 in first 5 weeks and the final week. (TIF)

S5 Fig Marginal posterior distribution of *ψ*_1_ (multiplicative term for contribution to infectious pressure from cattle) versus log *γ*_1_ (multiplicative factor contributing the infection pressure from infected farms) for the first 6 weeks of the 2001 UK outbreak. (TIF)

S6 Fig Marginal distribution of *ϕ*_2_ (exponential term for susceptibility of pigs) versus *ζ*_2_ (multiplicative factor for susceptibility of pigs relative to cattle) for the first six weeks of the Miyazaki outbreak. (TIF)

S7 Fig Expected number of undetected infections per county per week in the 2001 UK outbreak, weeks 1–16, as estimated under accrued information (y-axis) and complete information (x-axis). Those estimated using ‘accrued information’ only used data available at the week in question whereas those estimated under ‘complete information’ were estimated using all data from the outbreak.
(TIF)

S8 Fig Expected number of undetected infections per county per week in the 2010 Miyazaki outbreak as estimated under accrued (y-axis) and complete information (x-axis). Those estimated using ‘accrued information’ only used data available at the week in question whereas those estimated under ‘complete information’ were estimated using all data from the outbreak.
(TIF)

S9 Fig Predicted final total culls for weeks 1–12 for several control strategies for the UK 2001 outbreak of foot-and-mouth disease. A) Predictions of final total culls at the first 12 weeks throughout the 2001 outbreak in UK under seven control strategies (week 1 represents 26 February 2001). Columns denoted ‘Ac’ (‘accrued’ information) represent those simulations generated using data from the time point in question, columns denoted ‘Co’ (complete information) represent simulations seeded using parameters estimated using all the data from the outbreak. B) Rankings are calculated from the mean of those distributions in (A). C) Proportion of times each control is chosen as the optimal intervention if draws are taken from distributions in (A).
(TIF)

S10 Fig Predicted final total culls for weeks 13–28 for several control strategies for the UK 2001 outbreak of foot-and-mouth disease. A) Predictions of final total culls at weeks 13–28 throughout the 2001 outbreak in UK under seven control strategies (week 1 represents 26 February 2001). Columns denoted ‘Ac’ (‘accrued’ information) represent those simulations generated using data from the time point in question, columns denoted ‘Co’ (complete information) represent simulations seeded using parameters estimated using all the data from the outbreak. B) Rankings are calculated from the mean of those distributions in (A). C) Proportion of times each control is chosen as the optimal intervention if draws are taken from distributions in (A).
(TIF)

S11 Fig Predicted final total culls for weeks 1–11 for several control strategies for the 2010 outbreak of foot-and-mouth disease in Miyazaki, Japan. A) Predictions of final total culls at the first 11 weeks throughout the 2001 outbreak in UK under seven control strategies (week 1 represents 27 April 2010). Columns denoted ‘Ac’ (‘accrued’ information) represent those simulations generated using data from the time point in question, columns denoted ‘Co’ (complete information) represent simulations seeded using parameters estimated using all the data from the outbreak. B) Rankings are calculated from the mean of those distributions in (A). C) Proportion of times each control is chosen as the optimal intervention if draws are taken from distributions in (A).
(TIF)

S1 File Algorithm for the calculation of the proportion of times each intervention is chosen as optimal. (DOCX)
