# Supplementary figures and images for: Real-time decision-making during emergency disease outbreaks
Source: PLoS Comput Biol. 2018 Jul 24;14(7):e1006202. doi: 10.1371/journal.pcbi.1006202 (PMC6075790; doi:10.1371/journal.pcbi.1006202)

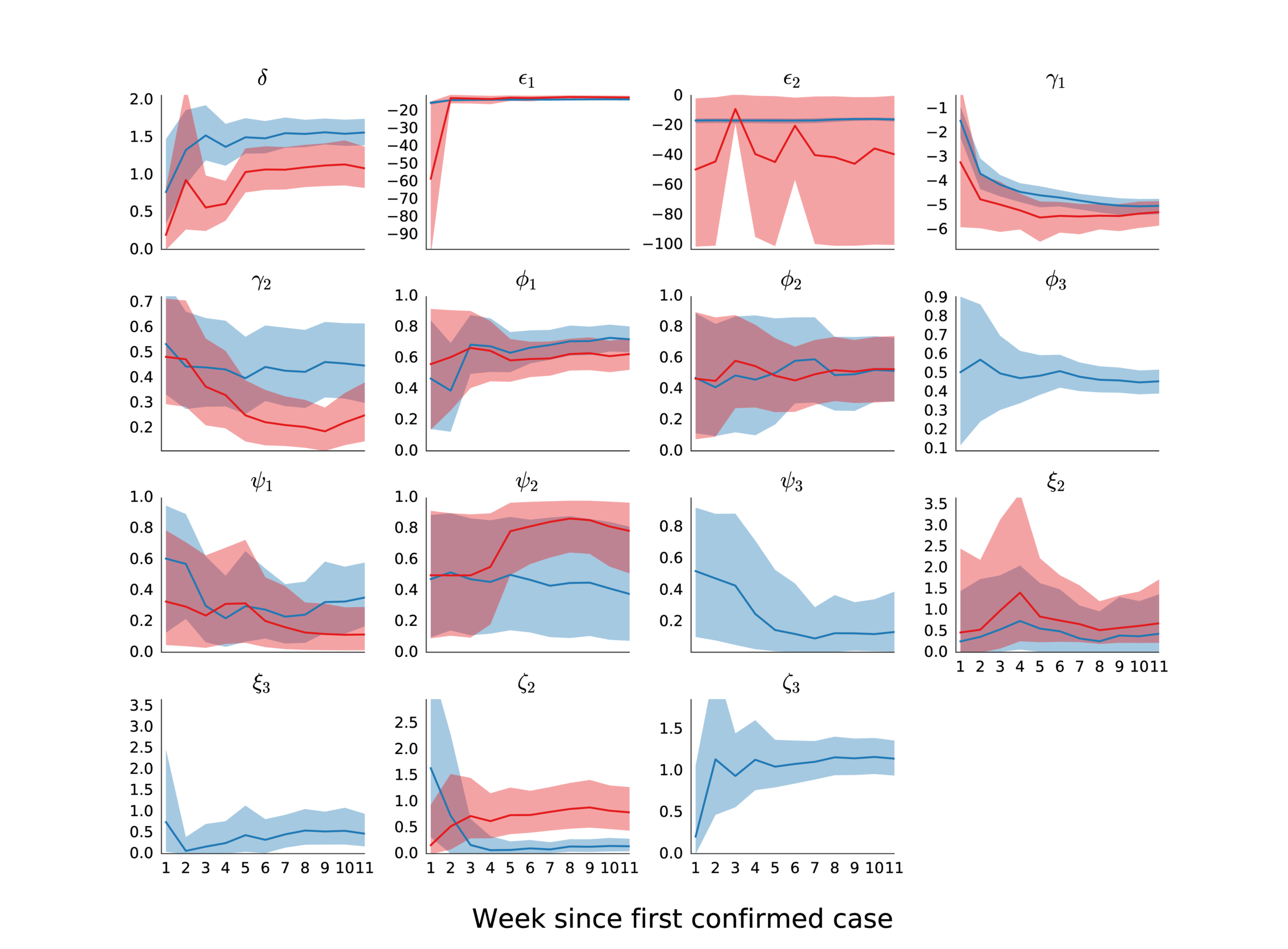

Supplement: S3 Fig — Distributions in red are estimated for the outbreak in Miyazaki blue for parameters the UK. Parameters shown on the log scale are γ1, ε1, and ε2. (TIF) [file pcbi.1006202.s003.tif]

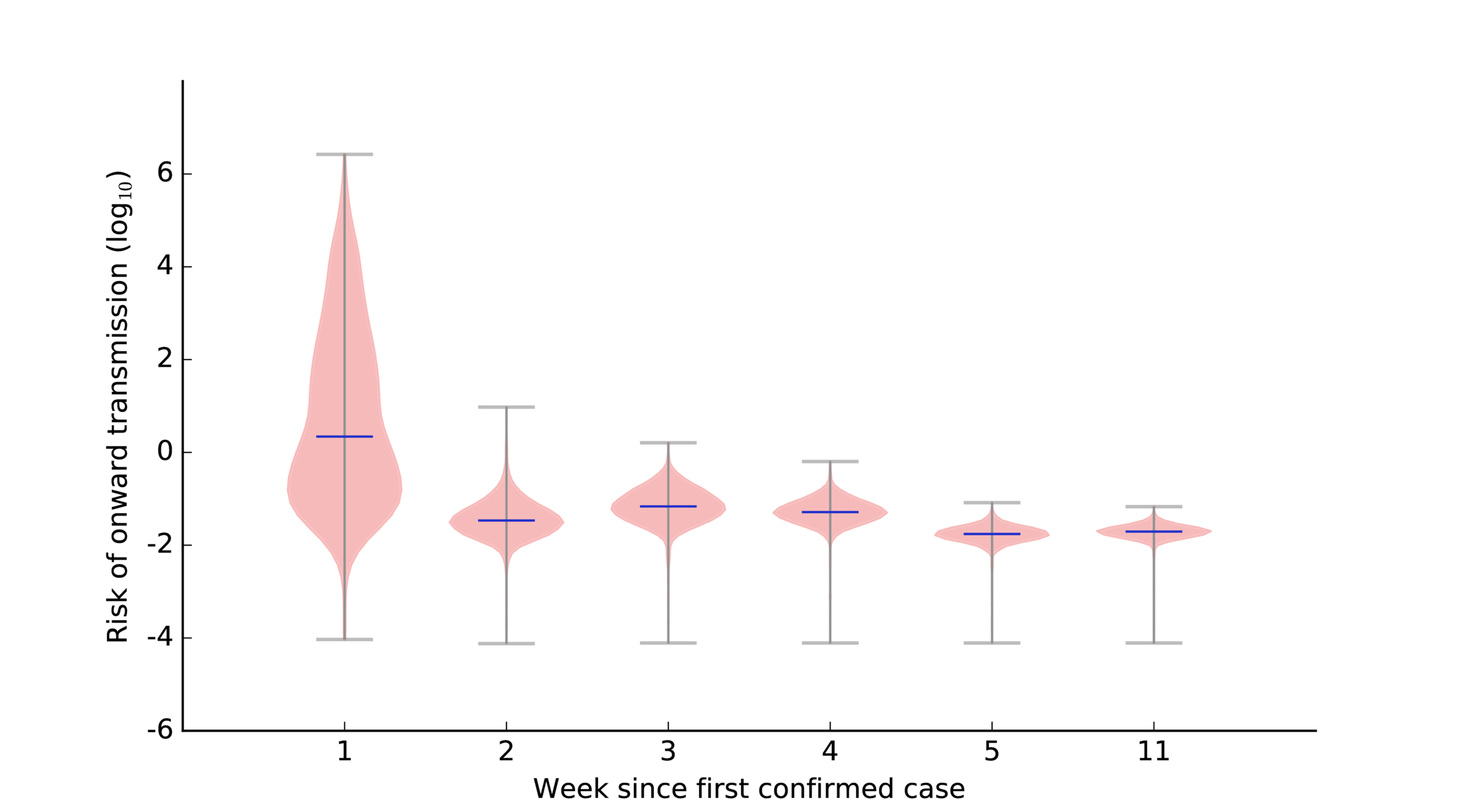

Supplement: S4 Fig — (TIF) [file pcbi.1006202.s004.tif]

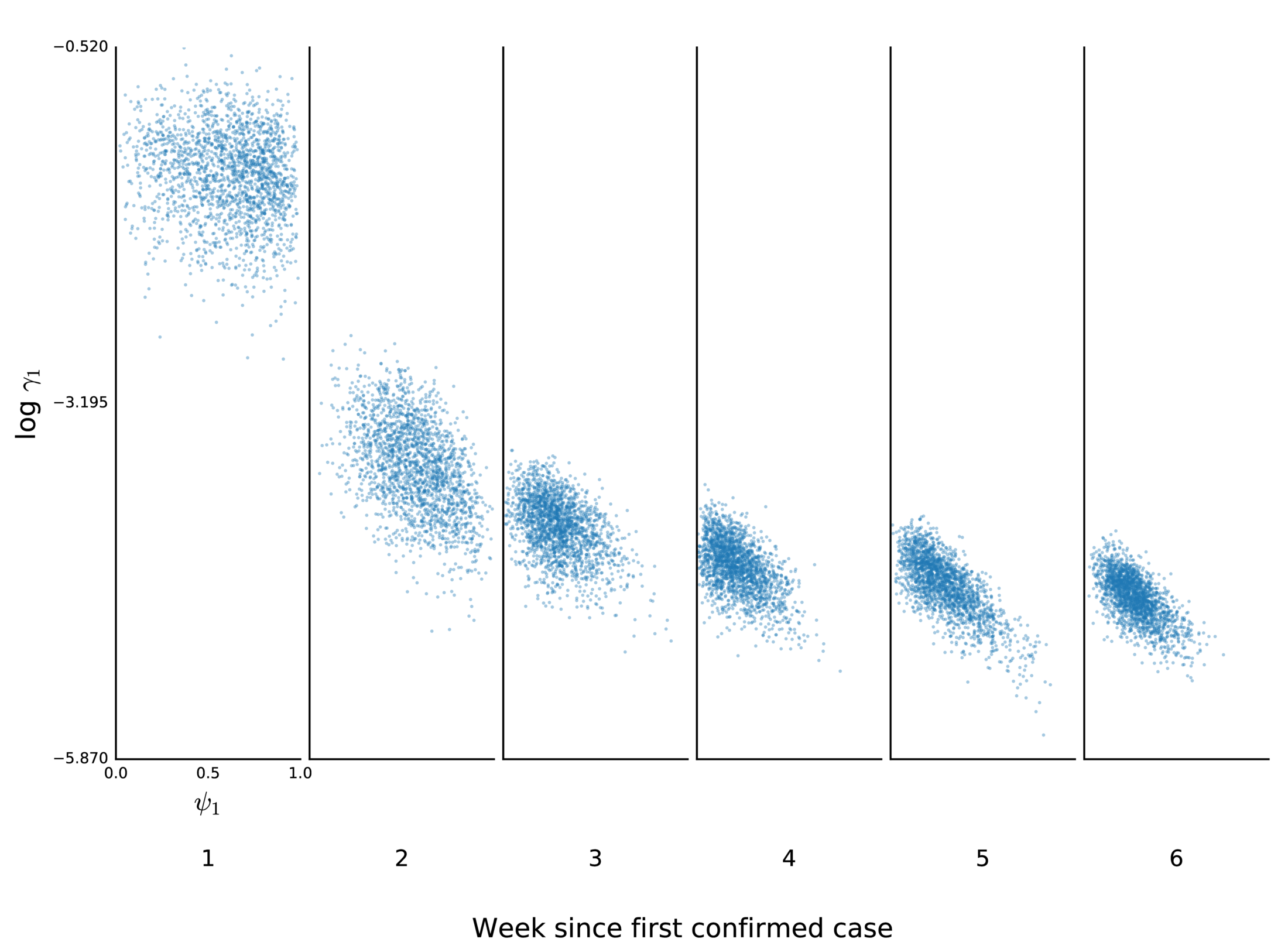

Supplement: S5 Fig — (TIF) [file pcbi.1006202.s005.tif]

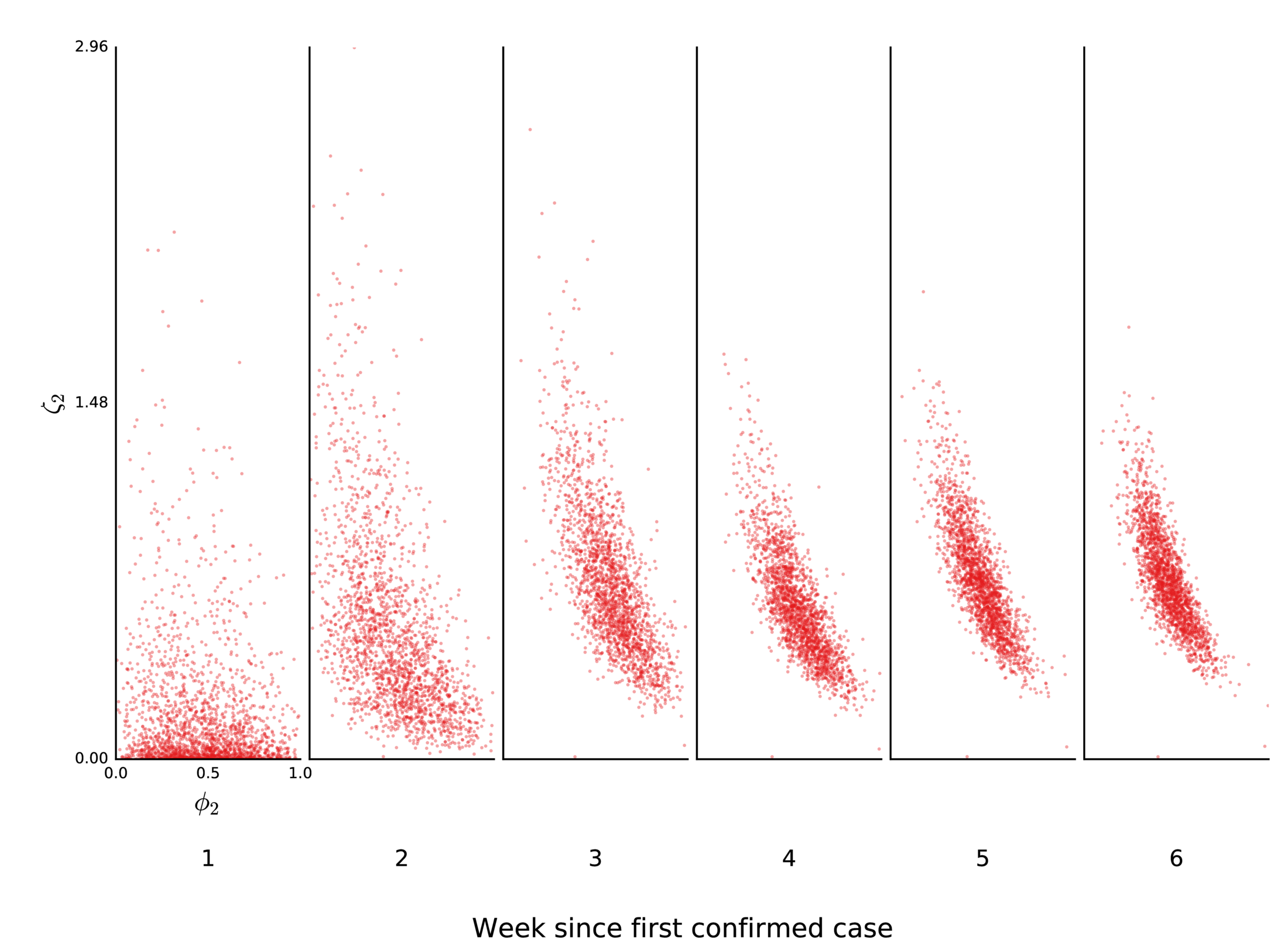

Supplement: S6 Fig — (TIF) [file pcbi.1006202.s006.tif]

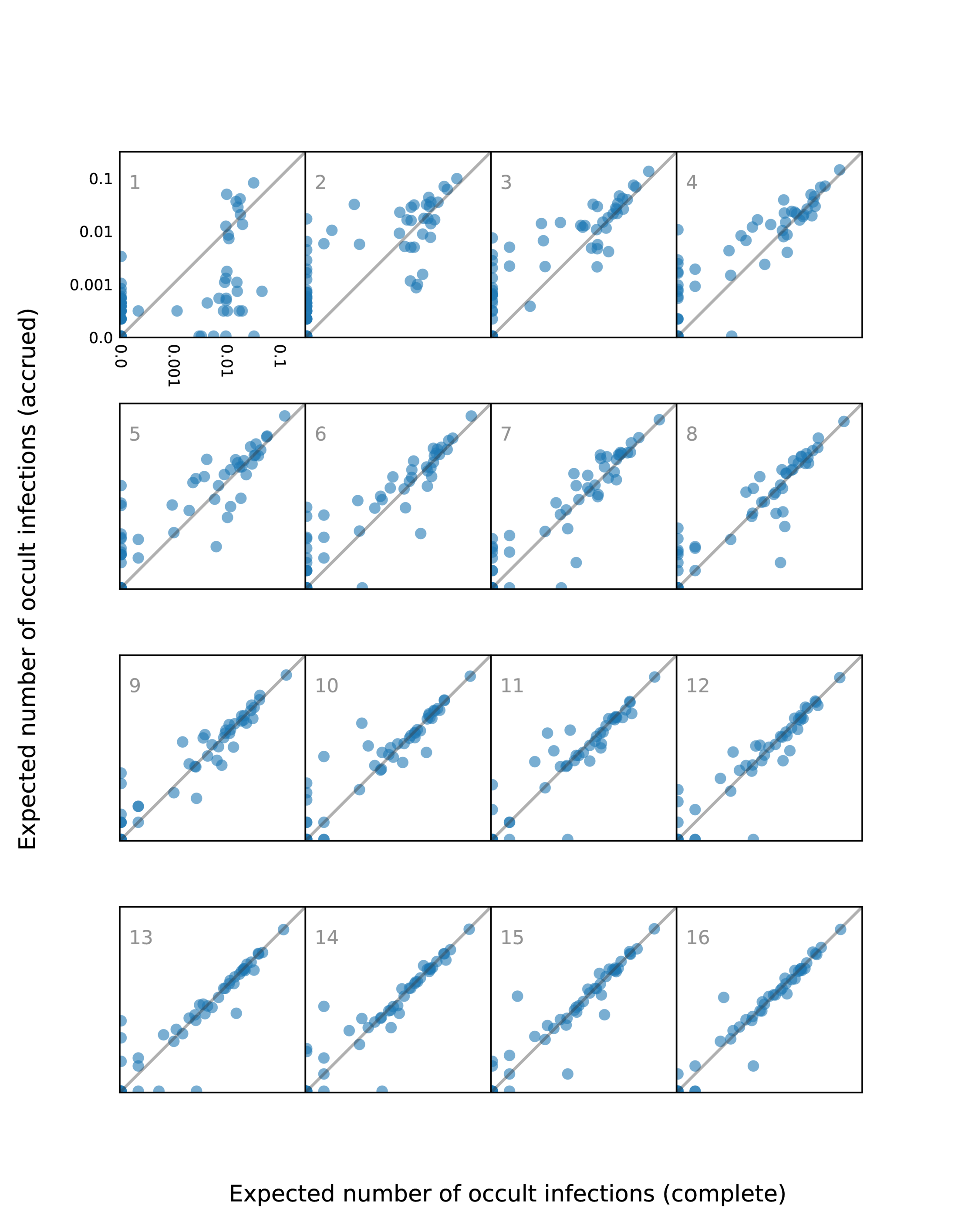

Supplement: S7 Fig — Those estimated using ‘accrued information’ only used data available at the week in question whereas those estimated under ‘complete information’ were estimated using all data from the outbreak. (TIF) [file pcbi.1006202.s007.tif]

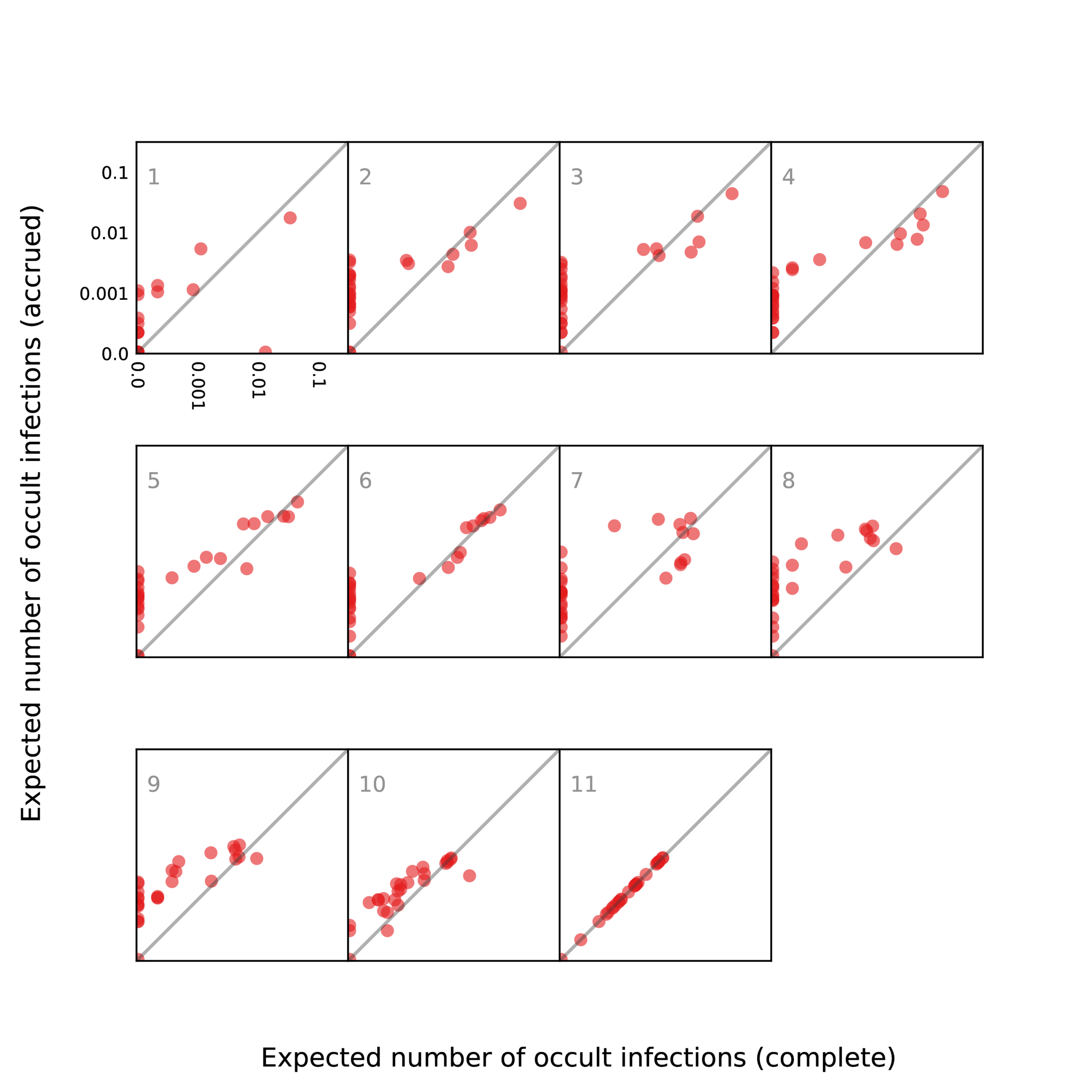

Supplement: S8 Fig — Those estimated using ‘accrued information’ only used data available at the week in question whereas those estimated under ‘complete information’ were estimated using all data from the outbreak. (TIF) [file pcbi.1006202.s008.tif]

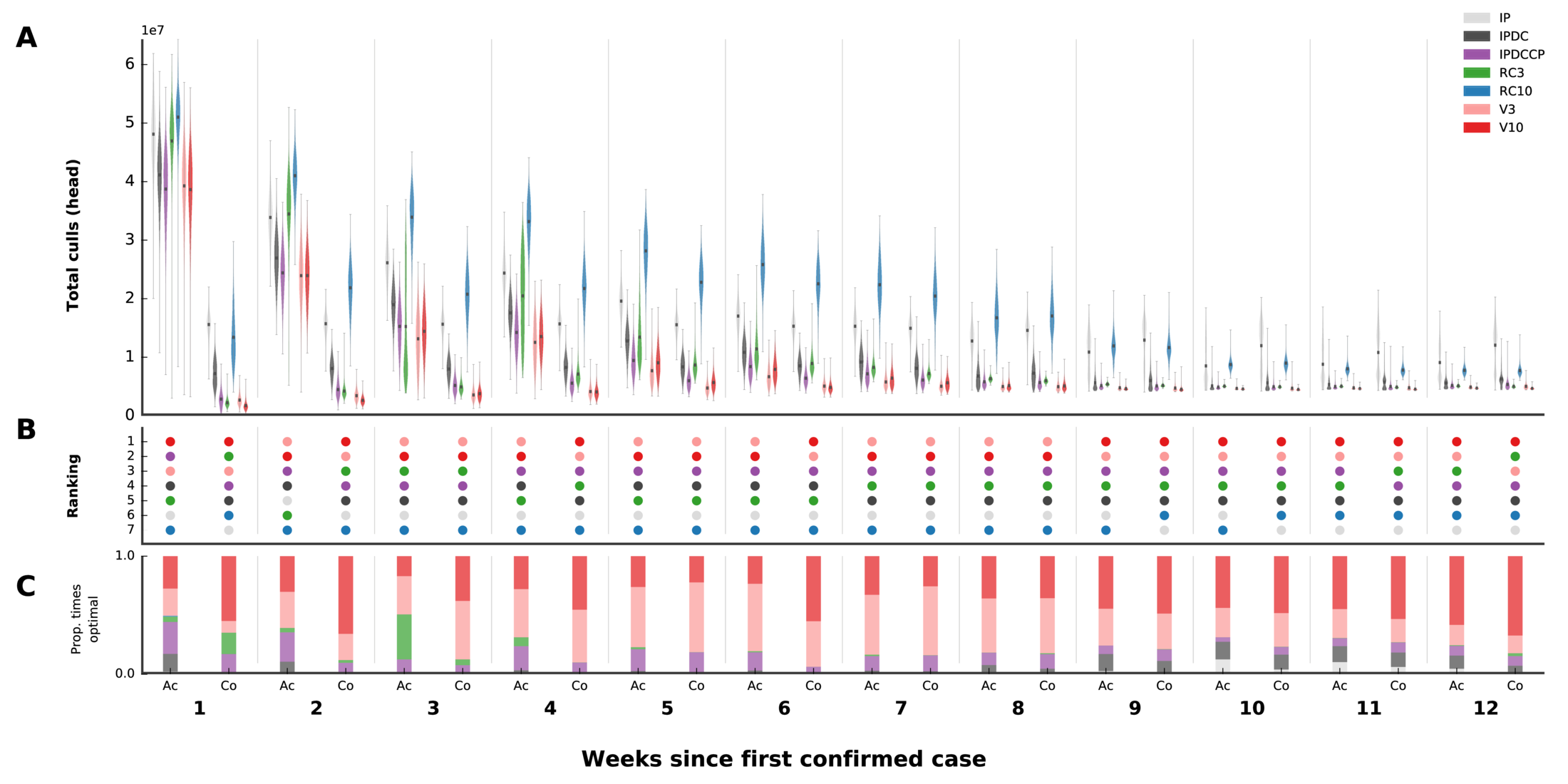

Supplement: S9 Fig — A) Predictions of final total culls at the first 12 weeks throughout the 2001 outbreak in UK under seven control strategies (week 1 represents 26 February 2001). Columns denoted ‘Ac’ (‘accrued’ information) represent those simulations generated using data from the time point in question, columns denoted ‘Co’ (complete information) represent simulations seeded using parameters estimated using all the data from the outbreak. B) Rankings are calculated from the mean of those distributions in (A). C) Proportion of times each control is chosen as the optimal intervention if draws are taken from distributions in (A). (TIF) [file pcbi.1006202.s009.tif]

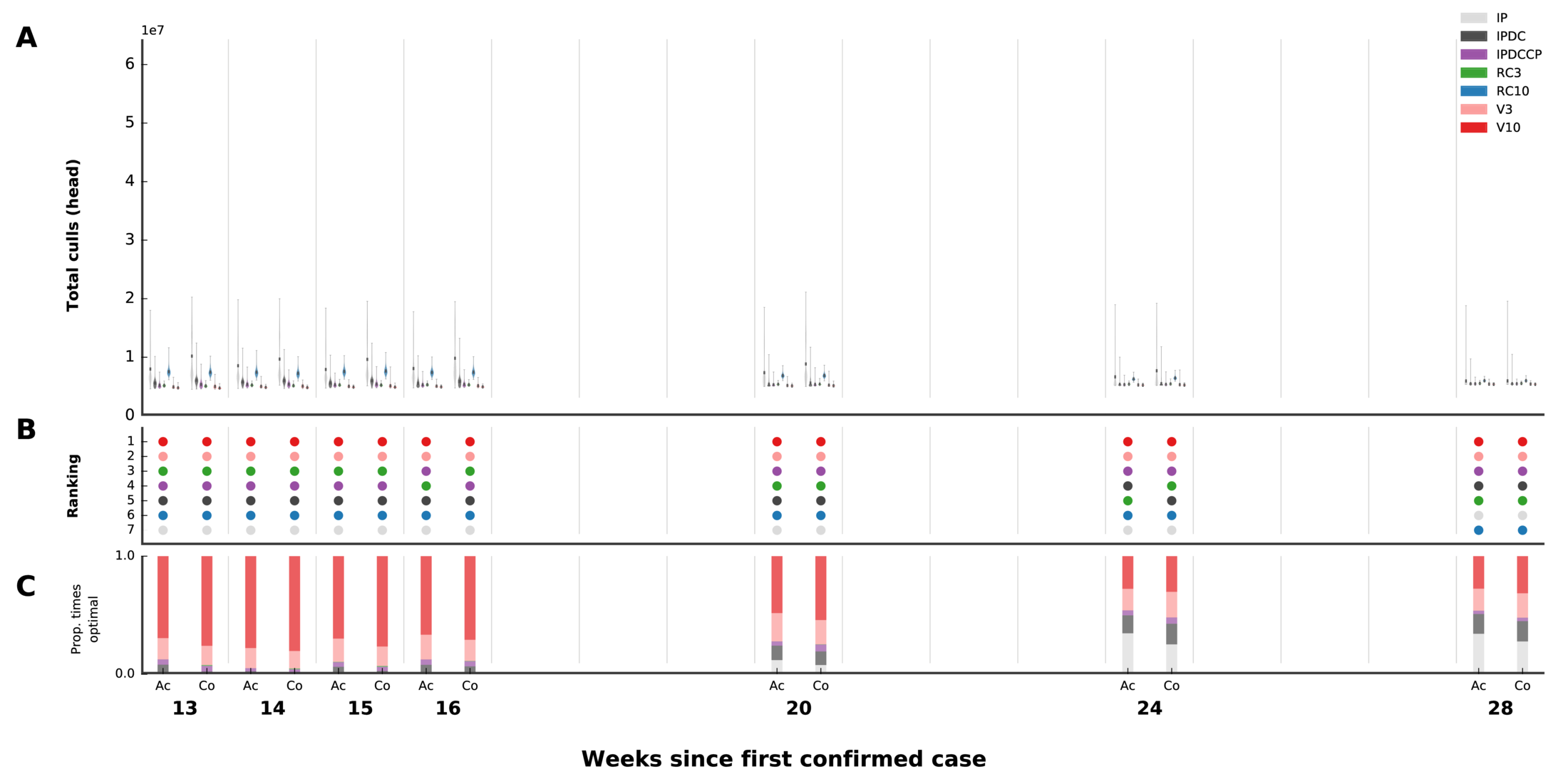

Supplement: S10 Fig — A) Predictions of final total culls at weeks 13–28 throughout the 2001 outbreak in UK under seven control strategies (week 1 represents 26 February 2001). Columns denoted ‘Ac’ (‘accrued’ information) represent those simulations generated using data from the time point in question, columns denoted ‘Co’ (complete information) represent simulations seeded using parameters estimated using all the data from the outbreak. B) Rankings are calculated from the mean of those distributions in (A). C) Proportion of times each control is chosen as the optimal intervention if draws are taken from distributions in (A). (TIF) [file pcbi.1006202.s010.tif]

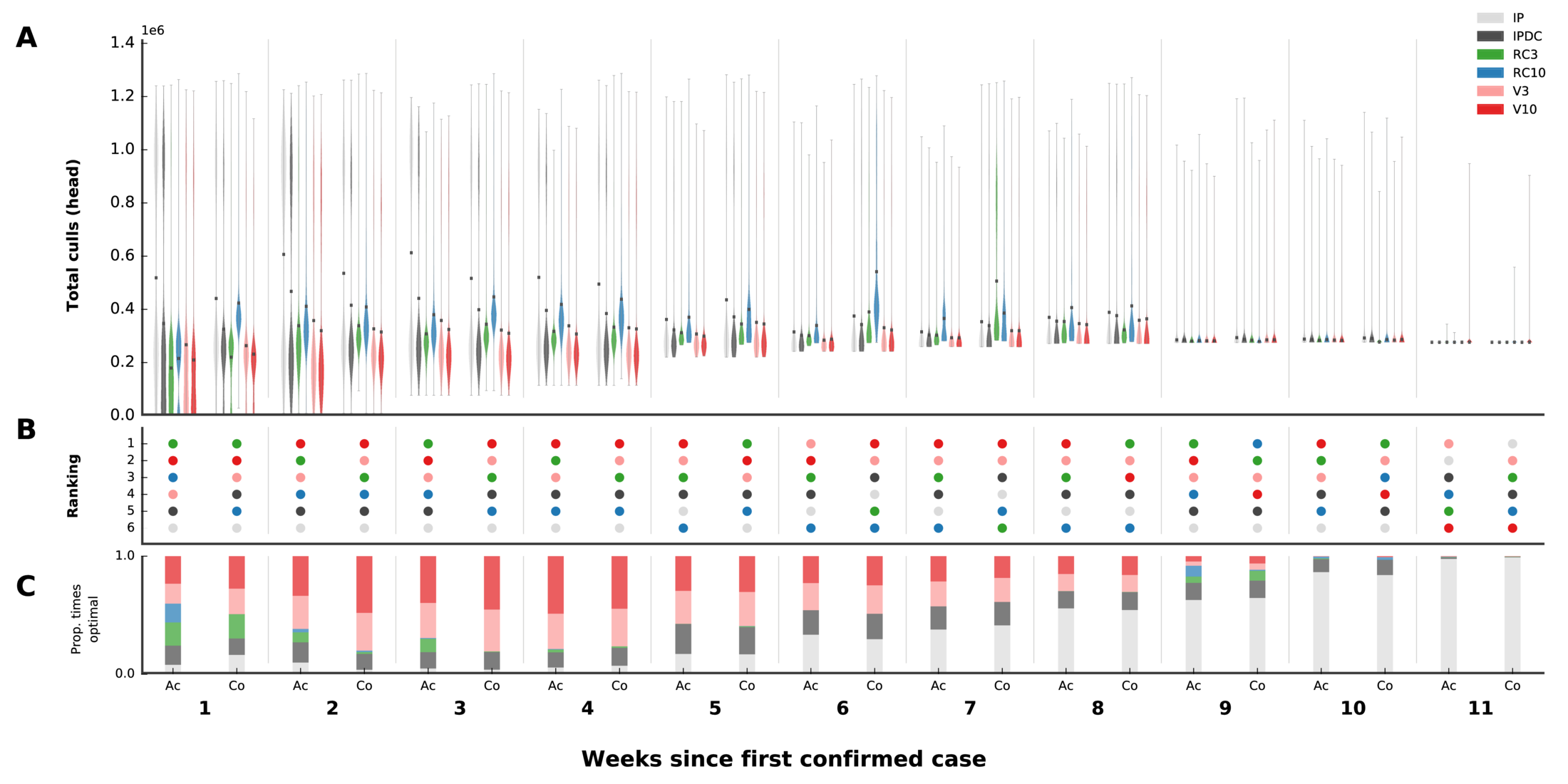

Supplement: S11 Fig — A) Predictions of final total culls at the first 11 weeks throughout the 2001 outbreak in UK under seven control strategies (week 1 represents 27 April 2010). Columns denoted ‘Ac’ (‘accrued’ information) represent those simulations generated using data from the time point in question, columns denoted ‘Co’ (complete information) represent simulations seeded using parameters estimated using all the data from the outbreak. B) Rankings are calculated from the mean of those distributions in (A). C) Proportion of times each control is chosen as the optimal intervention if draws are taken from distributions in (A). (TIF) [file pcbi.1006202.s011.tif]
