# Supplementary material for: Real-time decision-making during emergency disease outbreaks
Source: PLoS Comput Biol. 2018 Jul 24;14(7):e1006202. doi: 10.1371/journal.pcbi.1006202 (PMC6075790; doi:10.1371/journal.pcbi.1006202)
Supplement: S1 File — (DOCX) [file pcbi.1006202.s012.docx]

**File S1. Algorithm for the calculation of the proportion of times each action is chosen as optimal.**

Data for stacked bar charts (Figs 2C, 3C, S9C, S10C, S11C) illustrate the proportion of times each control action was chosen as optimal (or equal to optimal) when 1000 bootstrap samples from the distribution of total culls were drawn from the numerical output of forward simulations at each point in time. Such proportions were calculated in the following manner.

For the distribution of total culls at time *t*, *X_t_*, do the following (repeat for all time steps):

- Initialize: $S_{t}^{a}=0 \forall a\in\left\{ IP, IPDC, IPDCCP \left( for UK only \right), RC3, RC10, V3, V10 \right\},\forall t$
- Repeat 1000 times:

1. Draw one value, $x_{t}^{a}$, randomly from the output of forward simulations from each control action $X_{t}^{a}$ at time *t*.
2. Rank the values $x_{t}^{a}$ drawn for each control action, and add 1 to the score for the control action that has the minimum (optimal) value, $S_{t}^{a}=S_{t}^{a}+1$ where $k=arg\min_{a} x_{t}^{a}$ (*k* may be a set of values if ties occur).

- Calculate the proportion of times each control action was drawn as the optimal (or equal to optimal) action from the final scores, $P_{t}^{a}=\frac{S_{t}^{a}}{\sum_{t} S_{t}^{a}}$.
